# 1262. Dolutegravir Plus 3TC In Antiretroviral Experienced Adults: Immunovirological Outcomes In a Multicenter Retrospective Cohort In Spain

**DOI:** 10.1093/ofid/ofac492.1093

**Published:** 2022-12-15

**Authors:** Luis Buzón-Martín, Carlos Dueñas Gutierrez, José Antonio Iribarren, Ignacio de los Santos, Alberto Diaz-De Santiago, Miguel Ángel Morán-Rodríguez, Guillermo Pousada, Claudia González Rico, Estela Moreno García, Eva Ferreira, Alicia Iglesias Gómez, Cristina Martín gómez, Julia gómez Barquero, Miguel V Egido Murciano, Jesús Troya

**Affiliations:** Hospital Universitario de Burgos, burgos, Castilla y Leon, Spain; Hospital Clínico de Valladolid, Valladolid, Castilla y Leon, Spain; Hospital Universitario Donostia, San Sebastian, Pais Vasco, Spain; Hospital Universitario de la Princesa, Madrid, Madrid, Spain; Puerta de Hierro University Hospital, Madrid, Madrid, Spain; Hospital Universitario de Araba, Vitoria, Pais Vasco, Spain; Hospital Álvaro Cunqueiro, Vigo, Galicia, Spain; Hospital Universitario Marqués de Valdecilla, Santander, Cantabria, Spain; Hospital Universitario de Navarra, Pamplona, Navarra, Spain; Hospital General de Segovia, Segovia, Castilla y Leon, Spain; Hospital Universitario de Salamanca, Salamanca, Castilla y Leon, Spain; Hospital Virgen de la Concha, zamora, Castilla y Leon, Spain; Hospital Rio Hortega, Valladolid, Castilla y Leon, Spain; Hospital San Jorge, Huesca, Aragon, Spain; Hospital Universitario Infanta Leonor, Madrid, Madrid, Spain

## Abstract

**Background:**

Dolutegravir-based dual therapies for treating people living with HIV (PLHIV) are strategies strongly recommended in several practice guidelines. The safety and efficacy combination of Dolutegravir (DTG) plus 3TC as a switching strategy in virologically suppressed patients was demonstrated in the TANGO study. Wide multicenter real life data supporting this treatment is needed. The aim of the current study was to describe the efficacy in terms of immunovirological outcomes in patients treated with this antiretroviral combination.

**Methods:**

From November 1^st^ 2020 to August 1^st^ 2021, data from 1062 PLHIV collected from 13 Spanish institutions were recorded in a multicenter, retrospective study; After discarding cases in which relevant variables were missing, finally 694 cases underwent statistical analysis. Inclusion criteria were age >18 years, and to receive treatment with DTG/3TC as a switch strategy. Immunovirological impact of this strategy ( CD4+, CD8+ and CD4+/CD8+ cell count, as well as HIV plasma viral load through weeks 24, 48 and 96 of follow up) was evaluated using multivariable mixed models where the individual was considered as a random effect. Sex and age were added as demographic covariables.

**Results:**

78% of patients were men, 16% had been previously diagnosed of AIDS. Mean age was 48 years old. Mean CD4 T cell count nadir was 300 cell/ml (160-480). We found a significant increase in CD4+ counts at 24, 48 and 96 weeks after switching drug strategy. We also detected a small increase in the CD4+/CD8+ count rate at 48 and 96 weeks. No significant change was found in CD8+ count. No differences were found on behalf of sex, between backbone drugs (73% switched from a TDF/FTC backbone and 26.9% from ABC/3TC) or amongst the different third agents used (62% switched from integrase inhibitors). We identified a strong and negative effect of having AIDS in the CD4+ count. However, this effect did not interact with the effect of switching drug strategies.

CD4, CD8 and CD4/CD8 after switch at 24, 48 and 96w

**Figure:**
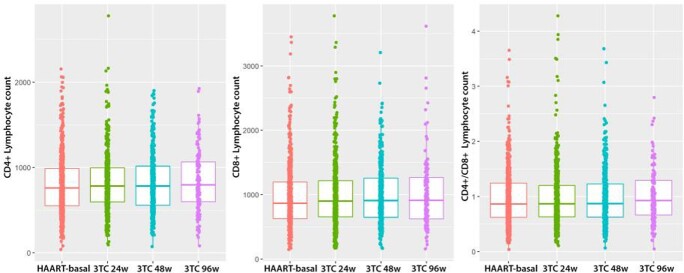

Log OR

**Figure:**
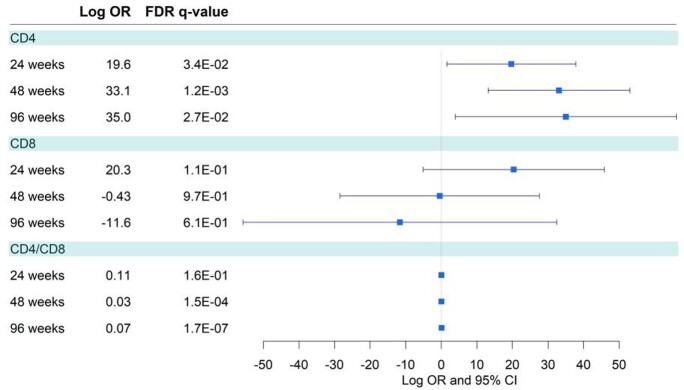

**Conclusion:**

PLHIV who, being virologically suppressed, switched to dual therapy based on DTG/3TC, experienced a statistically significant increase of CD4+ T cell count at weeks 24, 48 and 96, as well as an increase in CD4/CD8 T cell ratio, as well as high efficacy in terms of viral replication suppression, independently of the stage of HIV infection.

**Disclosures:**

**Luis Buzón-Martín, MD**, ViiV, GILEAD, Jannsen, MSD: Lecture fees **Carlos Dueñas Gutierrez, MD**, ViIV, GILEAD, Jannsen, MSD: Lecture fees **Jesús Troya, Staff**, ViiV, GILEAD, Jannsen, MSD: Lecture fees.

